# The associations of adherence to the Mediterranean diet with chronic dizziness and imbalance in community-dwelling adults: KNHANES 2019–2021

**DOI:** 10.1186/s12967-024-05295-4

**Published:** 2024-05-31

**Authors:** Seong-Hae Jeong, Eun Ji Kim, Eunjin Kwon, Ji-Soo Kim, Sukyoung Jung

**Affiliations:** 1 Department of Neurology, Chungnam National University Hospital, Daejeon, South Korea; 2 Department of Neurology, Chungnam National University School of Medicine, Daejeon, South Korea; 3 Department of Neurology, Dizziness Center, and Clinical Neuroscience Center, Seoul National University Bundang Hospital, Seongnam, South Korea; 4 Department of Neurology, Seoul National University College of Medicine, Seoul, South Korea; 5 Department of Health Care Policy Research, Korea Institute for Health and Social Affairs, 370 Sicheong-daero, Sejong, 30147 South Korea

**Keywords:** Imbalance, Dizziness, Chronic, Diet, Mediterranean

## Abstract

**Background:**

Dizziness and vertigo rank among the top 10 reasons for emergency and clinical referrals to neurologists. Chronic dizziness and imbalance not only reduce quality of life, but also increase mortality. While the Mediterranean diet has long been considered beneficial for human and planetary health, its effects on chronic dizziness or imbalance are understudied. We investigated the associations of adherence to the Mediterranean diet with chronic dizziness and imbalance.

**Methods:**

This study used data from the Korea National Health and Nutrition Examination Survey 2019–2021 and included 4,183 adults aged 40 years and older with complete information from diet, dizziness, and neurotology questionnaires. The alternate Mediterranean diet score (aMed) for nine food groups was calculated from 24-hour dietary recall data. Based on questionnaire responses, chronic dizziness was categorized as either isolated or chronic dizziness with imbalance, characterized by a cluster of difficulties maintaining a standing position, walking, or falling.

**Results:**

In a multivariable-adjusted model, the prevalence of chronic imbalance was lower in the top aMed tertile than in the bottom tertile (OR 0.37; 95% CI, 0.18–0.74; *p*-trend = 0.01). Among the individual aMed components, the intake of whole grains and nuts exhibited an inverse relationship with chronic imbalance (OR 0.50; 95% CI, 0.27–0.93 for whole grains; OR 0.55; 95% CI, 0.31–1.01 for nuts). The aMed score was not associated with isolated chronic dizziness.

**Conclusions:**

Greater adherence to the Mediterranean diet may reduce chronic imbalance, particularly with an adequate intake of whole grains and nuts.

**Supplementary Information:**

The online version contains supplementary material available at 10.1186/s12967-024-05295-4.

## Introduction

Vertigo and dizziness are among the top 10 reasons for emergency room and clinical referrals to neurologists. Chronic dizziness and imbalance are known to cause a significant decrease in individuals’ quality of life, while also posing a higher risk of falls and mortality [1]. Effective management of these conditions is crucial to prevent additional complications and to enable individuals to perform daily activities safely and with ease. Similar to other chronic conditions, including heart disease, cancer, and diabetes, dizziness and imbalance may necessitate ongoing medical care [2]. When treating dizziness and imbalance, we must consider the contributing factors in patients and work to improve their condition.

A healthy diet can be a cost-effective strategy to treat chronic dizziness and imbalance. The Mediterranean diet encourages consumption of monounsaturated fat, plant proteins, whole grains, and fish, together with moderate alcohol intake and limited consumption of red meat, refined grains, and sweets [3]. Therefore, the Mediterranean diet has long been recognized as a healthy diet and is associated with a reduced risk for many chronic diseases, including some neurological disorders [4]. Furthermore, the Mediterranean diet has received renewed attention for its potential environmental benefits [5].

Despite the clinical importance of chronic dizziness and balance problems, as well as the potential beneficial role of the Mediterranean diet in neurological diseases, to our knowledge, there is currently no research focusing on the Mediterranean diet. A case-control study conducted in Turkey showed that patients with dizziness or vertigo have undesirable dietary habits including skipping meals, lower consumption of water, carotene, and vitamin K, and higher consumption of bread than controls [6]. This knowledge gap highlights the need for further research to investigate the potential link between the Mediterranean diet and chronic dizziness and imbalance. Understanding this relationship could provide valuable insights into preventive measures and treatment options for individuals experiencing these symptoms. This study aimed to examine the associations of adherence to the Mediterranean diet with chronic dizziness and imbalance in Korean adults (aged ≥ 40 years) using data from the Korea National Health and Nutrition Examination Survey (KNHANES) 2019–2021.

## Methods

### Study population

The Korea Disease Control and Prevention Agency (KDCA) established the KNHANES, a cross-sectional, ongoing, and nationally representative survey, to monitor the health and nutritional status of the non-institutionalized population in Korea [7]. The KNHANES uses a complex and multistage probability sampling design to select a representative sample of the Korean population. Data are collected through three surveys: a health interview, health examinations, and nutrition surveys. Details on the KNHANES are available on its website (https://knhanes.kdca.go.kr/knhanes/main.do).

**Fig. 1:**
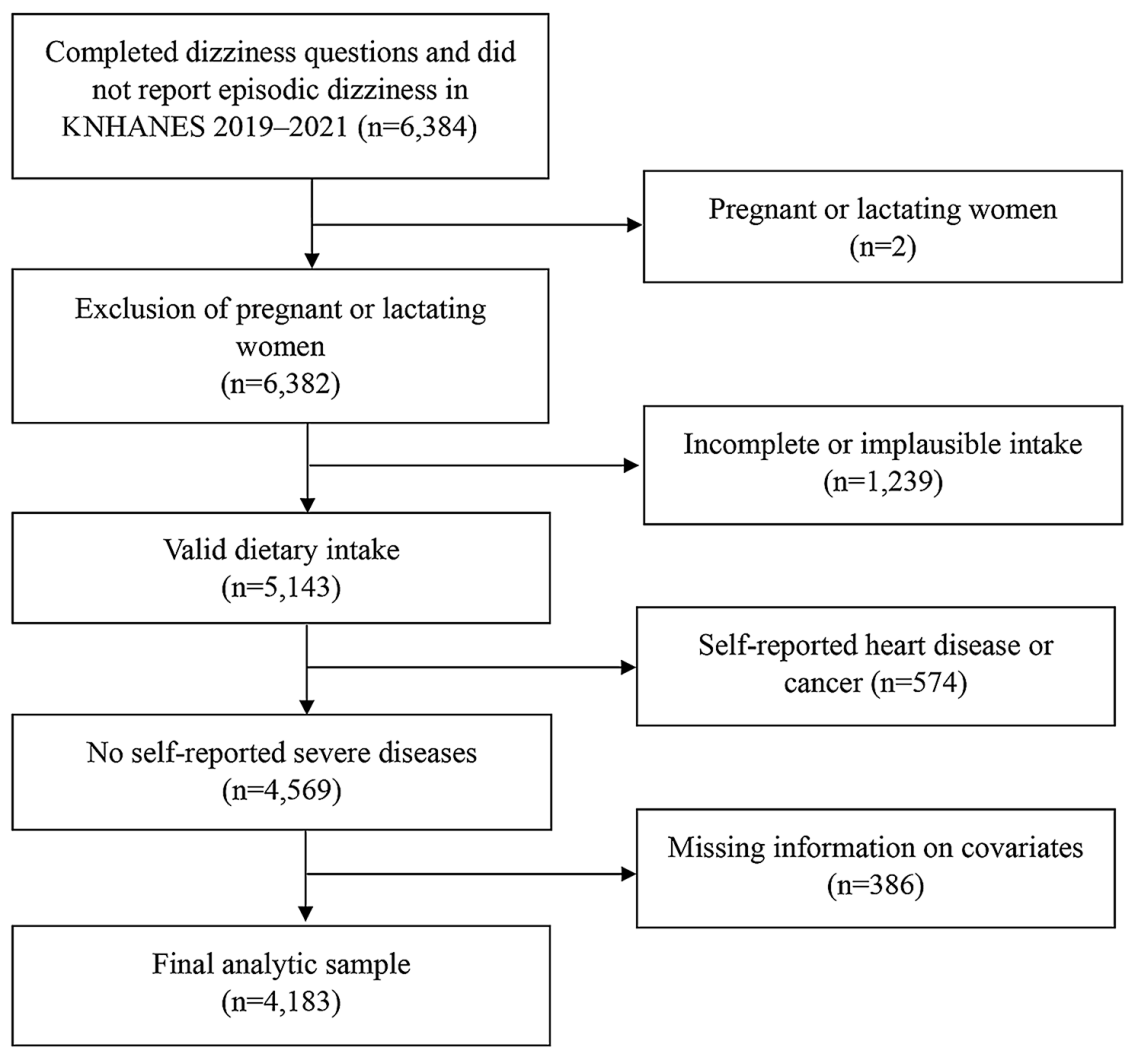
Study participant flowchart

Among 6,384 adults aged 40 years and older who completed the chronic dizziness questionnaire, we excluded participants if they had the following conditions: pregnancy or lactation (*n* = 2); incomplete or implausible energy intakes (< 500 kcal or > 5,000 kcal) (*n* = 1,239); self-reported coronary heart disease and cancer (*n* = 574); or missing information on sociodemographic variables (*n* = 386). The final analytic sample included 4,183 adults (1,852 men and 2,331 women) (Fig. 1).

### Assessment of the exposure: the alternate Mediterranean diet score

To collect detailed dietary information from survey participants, trained dieticians conducted a 24-hour dietary recall interview in participants’ homes 1 week after the health interviews and examinations. Participants reported the amounts (in units of volume), time, and place of eating of foods and beverages consumed during the past 24 h. The multiple-pass approach, with the assistance of a standard set of measuring guides, was applied to obtain accurate data on food recall. The daily intake of total energy and nutrients was estimated using the 10th Edition of the Korean Food Composition Table of the Rural Development Administration [8], and this information is publicly available on the KNHANES website.

The alternate Mediterranean diet (aMed) score was calculated using nine components: components for which consumption is encouraged (whole grains, vegetables, fruits, seafood, nuts, legumes, ratio of monounsaturated fatty acids to saturated fatty acids) and components for which consumption is limited (red and processed meat and alcohol) [9]. Sex-stratified median intake was used as a criterion. For the encouraged components, 1 point was assigned if the intake was above the median and 0 points otherwise. For the limited components, 1 point was assigned if the intake was below the median and 0 points otherwise. The total aMed score was calculated as the sum of all points from each component and ranged from 0 to 9, with a higher score indicating closer adherence to the Mediterranean diet.

### Assessment of chronic dizziness and imbalance

Chronic dizziness and imbalance were assessed using the KNHANES questionnaire. Chronic dizziness was defined as an affirmative answer to “Have you ever experienced dizziness or impaired balance in the past 12 months?” and “Have you ever felt chronically dizzy in the past 3 months or more?” [10]. Chronic dizziness with imbalance was defined as having chronic dizziness and an affirmative answer to at least one of the following questions: “Have you had difficulty maintaining a standing position in the past 3 months or more? (postural instability in standing)”, “Have you had difficulty walking in the past 3 months or more? (postural instability in walking)” or “Have you fallen down repeatedly in the past 3 months or more? (falling)” We defined those who had neither chronic dizziness nor chronic dizziness with impaired balance as the robust group (reference) [10] (Supplemental Fig. 1).

### Assessment of covariates

We used the following variables as covariates: age (years), sex (men or women), residential area (urban or rural), education level (less than high school graduate or high school graduate or higher), monthly household income (quartiles of equivalized household income), marital status (married or not), current smoking (yes or no), current drinking (yes or no), walking for exercise (yes or no), weight training (yes or no), perceived stress (yes or no), physician-diagnosed depression (yes or no), experience of tinnitus for more than 5 min (yes or no), the presence of hearing loss (yes or no), body mass index (BMI, kg/m^2^), and total energy intake (kcal/day) [10].

Walking for exercise was defined as walking five or more days a week for at least 30 min per session. Weight training was defined as engaging in weight training two or more days a week. Perceived stress was defined as experiencing stress moderately or severely based on self-reports. The experience of tinnitus was defined as an answer affirming that one had experienced a sense of ringing in one’s own ears for more than 5 min. The presence of moderate-to-profound hearing loss was defined as an unaided average pure tone hearing threshold level of 41 dB or greater. Pure-tone hearing levels were measured in a soundproof booth using an automatic audiometer (AD629; Interacoustics, Denmark). Standing height (cm) was measured on a stadiometer, and body weight (kg) was measured with a metric weight scale, with sample participants in light clothing. The BMI (kg/m^2^) was calculated as the ratio of measured weight to standing height squared [7].

### Statistical analysis

Based on previous studies, the required sample size was 12,149 participants, assuming a statistical power of 80%, a two-sided significance level of 0.05, and an odds ratio (OR) of 0.76 [11], with the proportion of chronic dizziness in the general population being 4.8% [12]. Our sample size (4,183 participants) may not be sufficient to detect the OR of 0.76 but may be sufficient to detect the OR of 0.39, which was the actual estimation of our study.

For categorical analyses, the aMed score was divided into tertile (T) groups, with T1 as the reference. The general characteristics of the study participants were described as the weighted means (standard errors [SEs]) for continuous variables and the weighted prevalence (SEs) for categorical variables by aMed tertile. The significance of differences among aMed tertiles was tested using analysis of variance for continuous variables and Rao-Scott chi-square test for categorical variables, respectively.

Multinomial logistic regression models were used to estimate ORs and their corresponding 95% confidence intervals (CIs) (isolated chronic dizziness vs. robust and chronic dizziness with imbalance vs. robust). Potential linear trends across aMed tertiles (*P* for trends) were determined by treating the median aMed score as a continuous variable. We presented two adjusted models: (1) an age and sex-adjusted model; and (2) a multivariable-adjusted model that additionally included education level, monthly household income, marital status, current smoking, current drinking, walking for exercise, weight training, perceived stress, depression, experience of tinnitus, hearing loss, BMI, and total energy intake. We further conducted stratified analyses for the association between aMed and chronic dizziness with or without imbalance by age (< 65 or ≥ 65 years) and sex (men or women). For the analysis, we used the PROC SURVEY procedures in SAS software (version 9.4, SAS Institute Inc., Cary, NC, USA) and applied survey weights accounting for the complex sampling design of the KNHANES. All tests were two-sided, and the level of significance was set at 0.05.

## Results

### Participant characteristics

Table 1 shows the characteristics of study participants by aMed tertiles. Among 4,183 participants, the median aMed score was 2.5 in T1 and 6.0 in T3. A higher aMed score was associated with older age, being married, not currently smoking or drinking, more walking or weight training, and less perceived stress. Men were predominantly in T2, while women were more evenly distributed across T1 and T3. The proportions of high school graduates and the tinnitus experience were higher in T2 than in T1 or T3. A higher aMed score was associated with higher intakes of energy and most macronutrients and micronutrients, except for carbohydrates and fats.

**Table 1:** Demographic and lifestyle characteristics of study participants by alternate Mediterranean diet score tertiles, KNHANES 2019–2021 (*n* = 4,183)

	aMed score tertiles			
	T1 (*n* = 1,423)	T2 (*n* = 1,186)	T3 (*n* = 1,574)	*P* value ^a^
Median aMed score (min, max)	2.5 (0.0–4.0)	4.3 (4.0–5.0)	6.0 (6.0–9.0)	
**Demographic**				
Age (years)	54.7 ± 0.4	55.9 ± 0.4	59.5 ± 0.4	< 0.0001
Age				
<65 years	78.0 (1.3)	78.0 (1.4)	68.2 (1.5)	< 0.0001
65 + years	22.0 (1.3)	22.0 (1.4)	31.8 (1.5)	
Sex				
Men	40.0 (1.4)	67.0 (1.4)	48.5 (1.4)	< 0.0001
Women	60.0 (1.4)	33.0 (1.4)	51.5 (1.4)	
Residential area				
Urban	83.3 (2.1)	83.9 (2.2)	83.0 (2.3)	0.8387
Rural	16.7 (2.1)	16.1 (2.2)	17.0 (2.3)	
**Socioeconomic**				
Education level				
Less than high school graduate	26.5 (1.5)	23.4 (1.6)	28.3 (1.4)	0.0276
High school graduate or above	73.5 (1.5)	76.6 (1.6)	71.7 (1.4)	
Household income				
Q1	16.0 (1.3)	13.3 (1.2)	15.8 (1.3)	0.0708
Q2	24.5 (1.5)	22.4 (1.5)	24.7 (1.6)	
Q3	29.9 (1.6)	27.6 (1.6)	27.5 (1.6)	
Q4	29.6 (1.8)	36.6 (2.2)	32.0 (2.1)	
Marital status				
Not married	8.0 (0.9)	5.9 (0.9)	4.2 (0.7)	0.0024
Married	92.0 (0.9)	94.1 (0.9)	95.8 (0.7)	
**Lifestyle**				
Current smoking status				
No	80.6 (1.4)	76.3 (1.5)	89.7 (0.9)	< 0.0001
Yes	19.4 (1.4)	23.7 (1.5)	10.3 (0.9)	
Current drinking status				
No	30.1 (1.4)	29.9 (1.5)	36.4 (1.5)	0.0011
Yes	69.9 (1.4)	70.1 (1.5)	63.6 (1.5)	
Walking for exercise ^b^				
No	64.3 (1.6)	63.0 (1.7)	57.1 (1.6)	0.0029
Yes	35.7 (1.6)	37.0 (1.7)	42.9 (1.6)	
Weight training ^c^				
No	82.1 (1.2)	76.8 (1.5)	75.1 (1.4)	0.0009
Yes	17.9 (1.2)	23.2 (1.5)	24.9 (1.4)	
**Mental health**				
Perceived stress				
No	74.5 (1.5)	78.9 (1.4)	81.9 (1.1)	0.0003
Yes	25.5 (1.5)	21.1 (1.4)	18.1 (1.1)	
Depression (physician-diagnosed)				
No	95.8 (0.6)	96.4 (0.7)	95.2 (0.6)	0.3873
Yes	4.2 (0.6)	3.6 (0.7)	4.8 (0.6)	
**Hearing ability**				
Experience of tinnitus				
No	93.0 (0.7)	89.6 (1.0)	90.7 (0.8)	0.0134
Yes	7.0 (0.7)	10.4 (1.0)	9.3 (0.8)	
Hearing loss				
No	86.6 (1.0)	86.9 (1.2)	85.2 (1.1)	0.4969
Yes	13.4 (1.0)	13.1 (1.2)	14.8 (1.1)	
Body mass index (kg/m^2^)	24.2 ± 0.1	24.5 ± 0.1	24.8 ± 0.1	0.1068
**Daily nutrient intake**				
Energy (kcal/day)	1676 (23)	1800 (24)	1940 (20)	< 0.0001
Carbohydrates (%kcal/day)	63.9 (0.4)	63.9 (0.4)	63.4 (0.3)	0.3976
Protein (%kcal/day)	14.6 (0.2)	15.4 (0.2)	15.9 (0.1)	< 0.0001
Fats (%kcal/day)	21.4 (0.3)	20.7 (0.3)	20.7 (0.2)	0.1848
Fiber (g/day)	20.9 (0.3)	26.9 (0.4)	34.7 (0.4)	< 0.0001
Calcium (mg/day)	417.6 (7.6)	490.9 (9)	587.9 (9.4)	< 0.0001
Sodium (mg/day)	2695.3 (49.7)	3294 (63.2)	3750.9 (56.9)	< 0.0001
Potassium (mg/day)	2247.8 (33.1)	2750.4 (40.6)	3391.8 (40.5)	< 0.0001
Magnesium (mg/day)	243.1 (3.4)	309.4 (4)	386.8 (4.1)	< 0.0001
Iron (mg/day)	7.7 (0.3)	9.5 (0.2)	11.5 (0.2)	< 0.0001
Zinc (mg/day)	8.4 (0.1)	9.9 (0.2)	11.4 (0.2)	< 0.0001
Vitamin A (μgRE/day)	296 (10.3)	374.3 (11.7)	486.0 (12.8)	< 0.0001
Vitamin E (mg/day)	5.1 (0.1)	6.6 (0.1)	8.3 (0.1)	< 0.0001
Vitamin B_1_ (mg/day)	1.0 (0.03)	1.1 (0.02)	1.2 (0.02)	< 0.0001
Vitamin B_2_ (mg/day)	1.3 (0.03)	1.4 (0.03)	1.7 (0.02)	< 0.0001
Niacin (mg/day)	10.0 (0.2)	11.7 (0.2)	13.2 (0.2)	< 0.0001
Vitamin C (mg/day)	54.4 (5.3)	60.0 (2.5)	87.6 (3.0)	< 0.0001
Vitamin D (mg/day)	2.0 (0.1)	3.2 (0.2)	4.2 (0.2)	< 0.0001

Abbreviation: KNHANES: Korea National Health and Nutrition Examination Survey; aMed: alternate Mediterranean diet score; RE: retinol equivalent; T: tertile

Note: All results are weighted and presented as mean and standard error (SE) for continuous variables and percentage and SE for categorical variables

^a^*P* values for differences by tertiles were obtained using general linear models for continuous variables and the Rao-Scott chi-square test for categorical variables

^b^ Walking for exercise was defined as walking five or more days a week for at least 30 min per session

^c^ Weight training was defined as weight training two or more days a week

### aMed in relation to chronic dizziness and imbalance

**Fig. 2:**
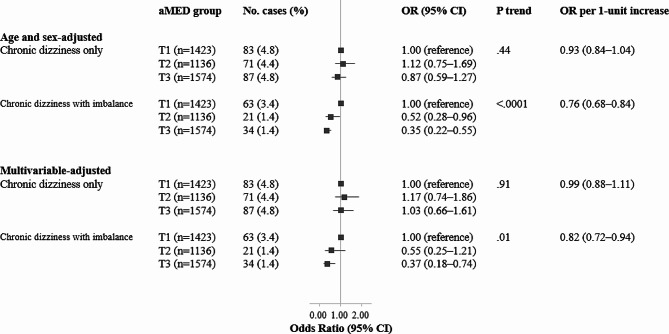
Odds ratios (95% confidence intervals) for chronic dizziness only and chronic dizziness with imbalance by alternate Mediterranean diet score tertiles, KNHANES 2019–2021 Abbreviation: KNHANES: Korea National Health and Nutrition Examination Survey; aMed: alternate Mediterranean diet score; T: tertile Note: Multinomial logistic regression models were used to estimate odds ratios and their corresponding 95% confidence intervals for chronic dizziness only and chronic dizziness with impaired balance compared to the robust group, with tertiles 2 and 3 of the aMed as the exposure variables. *P* for trends was determined by treating the median value of the aMed score as a continuous variable using multinomial logistic regression models. A multivariable-adjusted model was adjusted for age, sex, residential area, education level, monthly household income level, marital status, current smoking, current drinking, walking for exercise, weight training, perceived stress, depression, experience of tinnitus, hearing loss, body mass index, and total energy intake

Figure 2 presents the cross-sectional associations of the aMed score with isolated chronic dizziness and chronic dizziness with imbalance. After adjusting for age and sex, the participants in the highest aMed tertile had a 65% lower risk of chronic dizziness with imbalance than those in the lowest tertile (OR 0.35, 95% CI 0.22–0.55, *p*-trend < 0.0001). The inverse association remained significant after further adjustment for other variables related to sociodemographic characteristics, lifestyle factors, health, and total energy intake (OR 0.37, 95% CI 0.18–0.74, *p*-trend = 0.01). There was no significant association between the aMed score and isolated chronic dizziness.

### Individual components of the aMed score in relation to chronic dizziness and imbalance

**Fig. 3:**
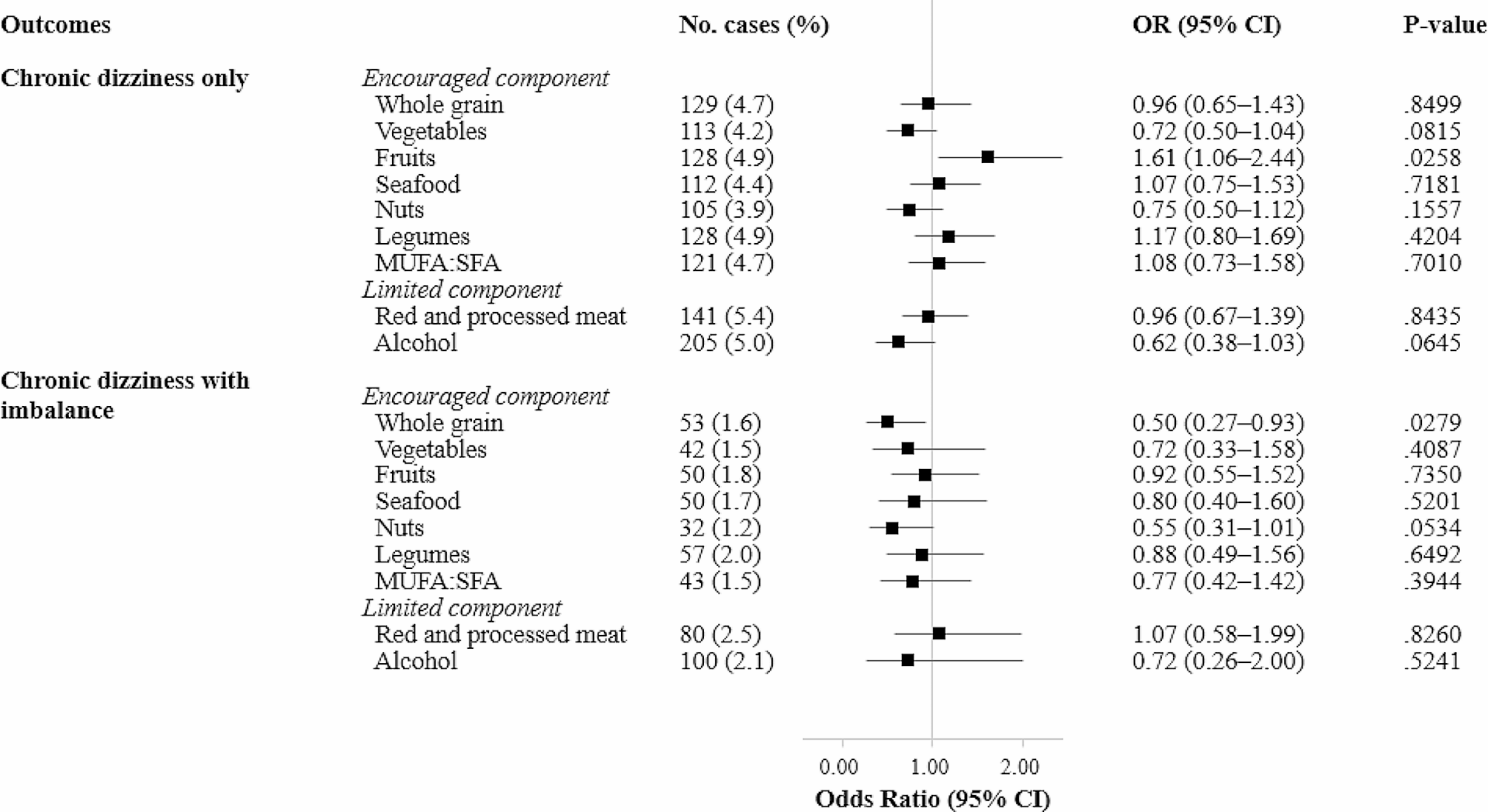
Associations of individual components of the alternate Mediterranean diet score with chronic dizziness only and chronic dizziness with imbalance Abbreviation: KNHANES: Korea National Health and Nutrition Examination Survey; aMed: alternate Mediterranean diet score; MUFA: monounsaturated fatty acid; SFA: saturated fatty acid Note: Multinomial logistic regression models were used to estimate odds ratios and their corresponding 95% confidence intervals for chronic dizziness only and chronic dizziness with impaired balance compared to the robust group, with the aMed as the exposure variables. Sex-specific median values were used (whole grain: 8.0 g/day for men, 10.4 g/day for women; vegetables: 324.4 g/day for men, 243.2 g/day for women; fruits: 26.7 g/day for men, 108.2 g/day for women; seafood: 55.3 for men, 34.8 for women; nuts: 0.59 g/day for men; 0.61 g/day for women; legumes: 9.7 g/day for men, 7.2 g/day for women; red and processed meat: 41.0 g/day for men, 19.6 g/day for women; alcohol: 0 g/day for both men and women). The multivariable-adjusted model was adjusted for age, sex, residential area, education level, monthly household income level, marital status, current smoking, current drinking, walking for exercise, weight training, perceived stress, depression, experience of tinnitus, hearing loss, body mass index, and total energy intake

Figure 3 shows the covariate-adjusted associations of individual aMed components with isolated chronic dizziness and chronic dizziness with imbalance. When analyzing individual components of the aMed score separately, the consumption of whole grains and nuts exhibited an inverse association with chronic dizziness with imbalance. In a multivariable-adjusted model, participants who consumed at least the median level of whole grains showed a 50% lower risk of chronic dizziness with imbalance (OR 0.50, 95% CI 0.27–0.93, *p*-trend = 0.03). For nuts, there was a suggestive inverse association between the aMed score and chronic dizziness with imbalance (OR 0.55, 95% CI 0.31–1.01, *p*-trend = 0.05). No significant associations were found between any of the individual components of the aMed score and isolated chronic dizziness.

### Subgroup analysis

Table 2 shows the stratified associations of the aMed score with chronic dizziness only and chronic dizziness with imbalance according to age and sex groups. When stratified by age, we observed an inverse association between aMed and chronic dizziness with imbalance in younger participants aged < 65 years (OR 0.16, 95% CI 0.05–0.53, *p* for trend = 0.0044), but not in older participants aged ≥ 65 years (OR 0.80, 95% CI 0.36–1.80, *p* for trend = 0.5013). When stratified by sex, we observed an inverse association between aMed and chronic dizziness with imbalance in women (OR 0.40, 95% CI 0.18–0.88, *P* trend = 0.0274), but not in men (OR 0.98, 95% CI 0.20–4.83, *P* trend = 0.6596). There was no significant association between the aMed score and isolated chronic dizziness across different age and sex groups.

**Table 2:** Odds ratios (95% confidence intervals) for chronic dizziness and chronic dizziness with imbalance by alternate Mediterranean diet score tertiles, stratified by age and sex groups, KNHANES 2019–2021

Subgroup	Alternate Mediterranean Diet Score (aMed)			*P* trend ^b^	OR per 1–unit increase
	T1	T2	T3		
**Age group**					
< 65 years (*n* = 2,696)					
Sample size	971	804	921		
No. of cases (chronic dizziness only) (%)	42 (3.6)	33 (3.1)	39 (3.5)		
No. of cases (chronic dizziness + imbalance) (%)	27 (2.5)	11 (1.2)	9 (0.5)		
Multivariable-adjusted OR (95% CI) ^a^					
Chronic dizziness only vs. robust	1.00 (reference)	0.96 (0.52–1.78)	0.93 (0.47–1.82)	0.8249	0.96 (0.81–1.14)
Chronic dizziness + imbalance vs. robust	1.00 (reference)	0.69 (0.28–1.74)	0.16 (0.05–0.53)	0.0044	0.77 (0.65–0.91)
≥ 65 years (*n* = 1,487)					
Sample size	452	382	653		
No. of cases (chronic dizziness only) (%)	41 (8.9)	38 (9.0)	48 (7.7)		
No. of cases (chronic dizziness + imbalance) (%)	36 (6.3)	10 (2.0)	25 (3.2)		
Multivariable-adjusted OR (95% CI) ^a^					
Chronic dizziness only vs. robust	1.00 (reference)	1.67 (0.82–3.40)	1.44 (0.76–2.72)	0.2923	1.07 (0.91–1.26)
Chronic dizziness + imbalance vs. robust	1.00 (reference)	0.33 (0.10–1.09)	0.80 (0.36–1.80)	0.5013	0.94 (0.77–1.15)
**Sex group**					
Men (*n* = 1,852)					
Sample size	466	718	668		
No. of cases (chronic dizziness only) (%)	14 (2.4)	28 (3.0)	18 (2.3)		
No. of cases (chronic dizziness + imbalance) (%)	11 (1.4)	8 (0.8)	6 (0.5)		
Multivariable-adjusted OR (95% CI) ^a^					
Chronic dizziness only vs. robust	1.00 (reference)	1.65 (0.51–5.37)	0.72 (0.16–3.31)	0.6134	0.83 (0.62–1.13)
Chronic dizziness + imbalance vs. robust	1.00 (reference)	0.35 (0.05–2.71)	0.98 (0.20–4.83)	0.6596	0.82 (0.56–1.19)
Women (*n* = 2,331)					
Sample size	957	468	906		
No. of cases (chronic dizziness only) (%)	69 (6.4)	43 (7.3)	69 (7.2)		
No. of cases (chronic dizziness + imbalance) (%)	52 (4.7)	13 (2.5)	28 (2.2)		
Multivariable-adjusted OR (95% CI) ^a^					
Chronic dizziness only vs. robust	1.00 (reference)	1.03 (0.59–1.81)	1.15 (0.74–1.80)	0.5350	1.02 (0.90–1.16)
Chronic dizziness + imbalance vs. robust	1.00 (reference)	0.64 (0.26–1.61)	0.40 (0.18–0.88)	0.0274	0.87 (0.75–1.01)

Abbreviations: KNHANES: Korea National Health and Nutrition Examination Survey; aMed: alternate Mediterranean diet score; T: tertile

Note: Multinomial logistic regression models were used to estimate odds ratios and their corresponding 95% confidence intervals for chronic dizziness only and chronic dizziness with impaired balance compared to the robust group, with tertile 2 and 3 of the aMed as the exposure variables

^a^ A multivariable-adjusted model was adjusted for age, sex, residential area, education level, monthly household income level, marital status, current smoking, current drinking, walking for exercise, weight training, perceived stress, depression, experience of tinnitus, hearing loss, body mass index, and total energy intake

^b^*P* for trends was determined by treating the median value of the aMed score as a continuous variable using multinomial logistic regression models

## Discussion

In this cross-sectional study of Korean adults, greater adherence to the Mediterranean diet, as reflected by a higher aMed score, was associated with reduced risk of chronic dizziness with imbalance after adjusting for sociodemographic and lifestyle variables. Among the nine components of the aMed score, only whole grains and nuts showed inverse associations with the presence of chronic dizziness with imbalance. In the subgroup analysis, these inverse associations between the aMed score and chronic dizziness with imbalance were observed in participants younger than 65 years of age and women. The Mediterranean dietary pattern was not found to be associated with chronic dizziness alone.

The interpretation of these findings should take into account the fact that chronic imbalance is a complex, multifactorial condition that causes instability and an increased risk of falling. Vestibular dysfunction in conjunction with other factors (e.g., musculoskeletal and visual impairment) is a major contributor to imbalance [13]. These systems can be disrupted by stroke, inflammation, trauma, toxicity, and neurodegenerative processes, which are overwhelmed by aging processes such as oxidative stress, and mitochondrial dysfunction, apoptosis, and disrupted Ca^2+^ homeostasis [14]. Although this interpretation is not definitive, it may be relevant that the encouraged components (e.g., whole grains, nuts, fruits, vegetables) in the Mediterranean diet contain various components, including polyphenols, vitamins, and essential fatty acids, which have been associated with reduced oxidative stress and inflammation [15, 16]. Thus, the Mediterranean diet may also be efficacious for chronic postural imbalance [10].

An alternative explanation is rooted in reports that adhering to the Mediterranean diet is associated with greater muscle strength and function and reduced risk of sarcopenia [17]. In a recent study, muscle mass decreased in patients with chronic dizziness and imbalance [18]. Therefore, it is possible that the Mediterranean diet can help reduce chronic dizziness and imbalance by mediating muscle mass. The Mediterranean diet’s muscle-protective properties may be linked to its balanced content of vitamins (such as vitamins E and C and carotenoids) and phytochemicals with antioxidant properties [19]. These nutrients protect the cells from damage due to reactive oxygen and nitrogen species (ROS/NRS) while maintaining healthy responses to low ROS and NRS levels [20]. In addition, polyphenols, dietary fibers, and monounsaturated and polyunsaturated fatty acids decrease inflammation by reducing pro-inflammatory mediators (e.g., C-reactive protein or interleukin-6) and modulating the gut microbiota [16]. The association between the Mediterranean diet, muscle mass, and chronic dizziness with imbalance needs to be examined further.

Our investigation revealed that a higher aMed score was associated with a lower prevalence of chronic imbalance. However, we did not find robust relationships between each of the components of the alternate Mediterranean diet score, except for whole grain and nut consumption. One possible explanation for this finding is that the impact of individual components may be small and only become apparent when these components are combined into an all-encompassing, unidimensional score. The Mediterranean diet may involve complex biological interactions between its different components, and the accurate identification of these interactions may require the use of large sample sizes [21]. Individual nutritional components are often analyzed by comparing their effects to the average risk associated with other nutrients. However, a dietary score can account for extreme levels of exposure (0 to 9) without the influence of other nutrients [22]. Whole grain cereals have been a part of the human diet since ancient times and can help prevent chronic diseases. They contain more fibers, proteins, vitamins, and inorganic salts and lower energy density than refined grains. Regular intake of whole grain cereals can significantly reduce the risk of chronic diseases and improve overall health [23]. A recent study conducted on the Korean population suggests that consuming nuts may lower the risk of low muscle strength among older adults [24]. Whole grain and nut consumption may be encouraged for people with chronic dizziness with imbalance in clinical settings. In our study, it is noteworthy that the ameliorative effects of the Mediterranean diet were not found to be significant in individuals with isolated chronic dizziness, but in those presenting with both chronic dizziness and imbalance.

Moving beyond food group intake, some studies have suggested that nutritional imbalances may cause dizziness/vertigo. A study conducted in Brazil found a link between benign paroxysmal positional vertigo (BPPV) and a diet high in carbohydrates and polyunsaturated fatty acids, along with low dietary fiber intake, in elderly individuals [11]. A recent meta-analysis suggested that vitamin D exerts secondary protective effects in BPPV [25]. In the context of dietary habits and patterns, a case-control study in Turkey, using a 24-hour dietary recall and a food frequency questionnaire, emphasized the importance of actively monitoring the irregular dietary habits and hydration of individuals with vertigo [6]. Gunes-Bayir et al. provide compelling evidence that not only what but also how we eat plays a critical role in managing dizziness and vertigo [6]. Partially aligning with this previous study, our study showed that patients experiencing chronic dizziness with imbalance may benefit from adhering to Mediterranean dietary patterns.

In our study, the inverse association between aMed and chronic dizziness with imbalance was only observed in younger adults and women. This phenomenon may be explained by differences in other attributes of the Mediterranean diet between age and sex groups, which were not available in the KNHANES (e.g., food environments or knowledge of the Mediterranean diet). Additionally, the association between disease and the Mediterranean dietary pattern is not simple and dependent on the type or the point of the disease process at which the diet is assessed. Further studies with more detailed information and repeated measures are necessary.

The present study has some strengths. To our knowledge, this study is the first to evaluate the association of the Mediterranean dietary pattern with chronic dizziness and imbalance; thus, a direct comparison of our findings with other reports in the literature was not possible. As a second strength, we used nationally representative data collected through a standardized protocol. Several limitations should be considered as well. First, a strong causal inference between the Mediterranean dietary pattern and chronic dizziness with imbalance could not be identified due to the cross-sectional nature of the study design. Second, there could have been misclassification of chronic dizziness with imbalance because self-reported data were used in its definition and a validation study of the chronic dizziness with imbalance questionnaire was not conducted. Due to the imprecise definition of “dizziness” and the study’s reliance on self-reported data rather than medically accurate and objective methods, the study cannot confirm whether the Mediterranean diet is related to specific conditions such as lightheadedness or vertigo. However, trained physicians and examinators administered the questionnaire using a standardized protocol, and such misclassification would be non-differential, which may bias the results towards the null. Further studies are needed with more accurate and validated methods for diagnosis of chronic dizziness with imbalance to establish a more precise link. Third, the direction or size of the association may be affected by unmeasured or unknown factors. Fourth, there could be measurement errors and underreporting issues in self-reported dietary assessments, including 24-hour dietary recall [26]. Due to day-to-day variation, the use of 24-hour dietary recall may not accurately reflect individuals’ usual intake, but it can be sufficient for assessing the population’s mean intake [27]. Furthermore, we used a predefined composite index to assess adherence to the Mediterranean diet, and thus it may not accurately reflect a balanced Mediterranean diet. Further studies using a food frequency questionnaire or clinical trials assigning a Mediterranean diet program to participants would be necessary to validate our findings. Finally, we focused on Korean adults aged 40 years and older, which may limit the generalizability to different study settings or other populations.

In conclusion, Korean adults who follow the Mediterranean dietary pattern, as measured by aMed, may have lower risk of chronic dizziness with imbalance than those who follow a less Mediterranean dietary pattern. Despite the cross-sectional design, our findings provide new knowledge of how the Mediterranean dietary pattern can be beneficial for balance disorders. Further research, especially using a prospective study design, is required to replicate our findings, provide more evidence for recommendations, and elucidate the underlying mechanisms.

### Electronic supplementary material

Below is the link to the electronic supplementary material.

Supplementary Material 1

## Data Availability

All data are publicly available at https://knhanes.kdca.go.kr/knhanes/main.do.
