# Supplementary material for: The associations of adherence to the Mediterranean diet with chronic dizziness and imbalance in community-dwelling adults: KNHANES 2019–2021
Source: J Transl Med. 2024 May 31;22:522. doi: 10.1186/s12967-024-05295-4 (PMC11140959; doi:10.1186/s12967-024-05295-4)
Supplement: Supplementary file 1 — Supplementary Material 1 [file 12967_2024_5295_MOESM1_ESM.docx]

**Supplementary material**

**Supplement to:** Seong-Hae Jeong, Eun Ji Kim, Eunjin Kwon, Ji-Soo Kim, Sukyoung Jung, The associations of adherence to the Mediterranean diet with chronic dizziness and imbalance in community-dwelling adults: KNHANES 2019–2021


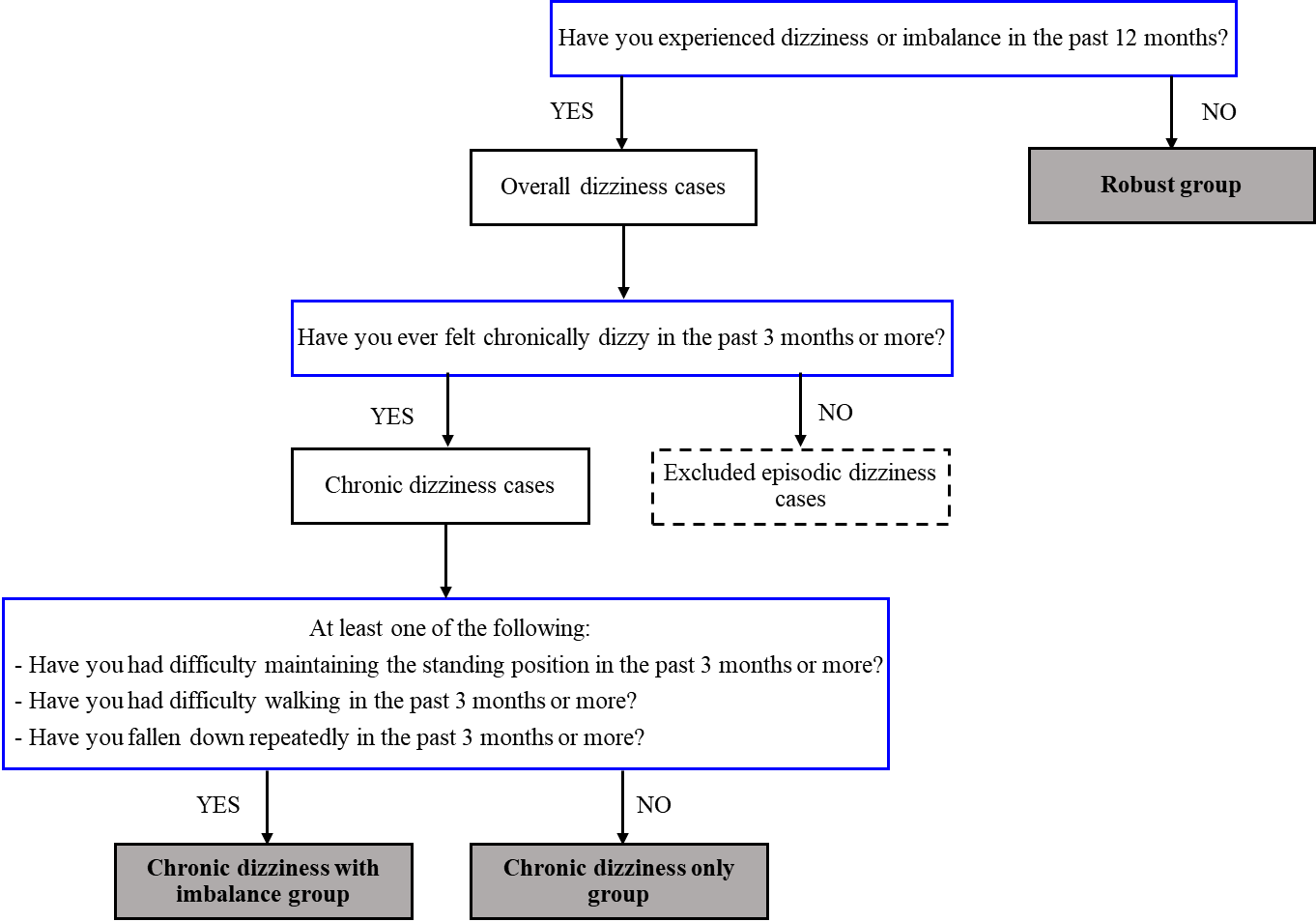


**Supplemental Figure 1. Flow chart of chronic dizziness and imbalance group classification**
